# Enhancing extracellular production of lipoxygenase in *Escherichia coli* by signal peptides and autolysis system

**DOI:** 10.1186/s12934-022-01772-x

**Published:** 2022-03-19

**Authors:** Cuiping Pang, Song Liu, Guoqiang Zhang, Jingwen Zhou, Guocheng Du, Jianghua Li

**Affiliations:** 1 National Engineering Research Center for Cereal Fermentation and Food Biomanufacturing, Jiangnan University, 1800 Lihu Road, Wuxi, 214122 Jiangsu China; 2 Science Center for Future Foods, Jiangnan University, Wuxi, 214122 China; 3 School of Biotechnology and Key Laboratory of Industrial Biotechnology, Ministry of Education, Jiangnan University, 1800 Lihu Road, Wuxi, 214122 Jiangsu China; 4 Engineering Research Center of Ministry of Education on Food Synthetic Biotechnology, Jiangnan University, 1800 Lihu Road, Wuxi, 214122 Jiangsu China; 5 Jiangsu Provisional Engineering Research Center of Food Synthetic Biotechnology, Jiangnan University, 1800 Lihu Road, Wuxi, 214122 Jiangsu China

**Keywords:** Autolysis, Lipoxygenase, Protein secretion, Signal peptide

## Abstract

**Background:**

Lipoxygenase (LOX) is a non-heme iron containing dioxygenase that is widely used to improve food quality and produce active drug intermediates and biodiesel. *Escherichia coli* is one of the most widely used host microorganisms for recombinant protein expression; however, its weak extracellular secretion ability precludes its effective production of recombinant proteins into the extracellular environment. To facilitate subsequent characterization and application of LOX, improving its secretion efficiency from *E. coli* is a major challenge that needs to be solved.

**Results:**

Several strategies were adopted to improve the extracellular secretion of LOX based on the signal peptides and cell wall permeability of *E. coli.* Here, we studied the effect of signal peptides on LOX secretion, which increased the secretory capacity for LOX marginally. Although surfactants could increase the permeability of the cell membrane to promote LOX secretion, the extracellular LOX yield could not meet the requirements of industrialization production. Subsequently, an autolysis system was constructed in *E. coli* based on the bacteriophage lysis gene ΦX174-E to enhance the production of extracellular proteins. Thus, the extracellular production of LOX was achieved and the content of inclusion bodies in the cell was reduced by optimizing cell lysis conditions. The extracellular LOX yield reached 368 ± 1.4 U mL^−1^ in a 5-L bioreactor under optimized lysis conditions that is, an induction time and temperature, and arabinose concentration of 5 h, 25 °C, and 0.6 mM, respectively.

**Conclusions:**

In this study, the different signal peptides and cell autolysis system were developed and characterized for extracellular LOX production in *E. coli*. Finally, the cell autolysis system presented a slight advantage on extracellular LOX yield, which also provides reference for other protein extracellular production.

**Supplementary Information:**

The online version contains supplementary material available at 10.1186/s12934-022-01772-x.

## Background

Lipoxygenase (EC. 1.13.11.12, LOX) mainly catalyzes the dioxygenation of polyunsaturated fatty acids with one or more *cis*, *cis*-1, 4-pentadiene units to produce hydroperoxy fatty acids [1], which are used in the food, chemical, and textile industries [2, 3]. Presently, most of the applied research is based on LOX extracted form soybean [4]. However, the complex isozymes and heterogeneity of raw materials make it challenging to control LOX quality to meet industrial requirements. With the rapid development of biotechnology, the endogenous and heterologous expression of LOX in microorganisms has been explored [5–7]. LOX produced by microorganisms has a higher purity (without isozyme) and specificity than that from soybeans [8]; however, some challenges persist, including insufficient expression levels and low enzyme activity.

*Escherichia coli* is one of the most commonly used microorganisms for recombinant protein expression [9, 10]. For example, the yield of recombinant *Pa*LOX is up to 23,850 U·mL^−1^ (264 mg pure protein/L culture fluid) in *E. coli* [11]. Compared to the accumulation of target protein in cells, extracellular secretion effectively simplifies downstream separation, avoids intracellular proteolytic degradation, and reduces protein aggregation [12]. Two main strategies for the use of *E. coli* to produce extracellular proteins have been reported: controllable cell lysis and transmembrane transport mediated by signal peptide [13]. Signal peptide-assisted recombinant protein secretion is the conventional method used for extracellular production. Extracellular protein production can be achieved by partially breaking or modifying the outer membrane or cell wall for permeability using physico-chemical methods (e.g., ultrasound/Triton X-100) or enzymatic treatments (e.g., lysozyme) [14, 15]. Interference with the cell wall peptidoglycan and membrane network using strain engineering can also effectively enhance the extracellular protein secretion of *E. coli* [16, 17].

The lysis gene ΦX174-E of the single-stranded DNA bacteriophage encodes a transmembrane pore-forming protein that can cleave the host cell membrane to induce the leakage of intracellular proteins [18]. Its transmembrane structure is formed by the interaction of lysis protein with targeted proteins involved in *E. coli* cell wall synthesis. Depending on the expression of bacterial phage lysis proteins, the cell membrane may be weakened or completely lysed, which is conducive to further protein release processes. The "empty cell" with almost intact cell walls is then formed after releasing its cellular contents to the outside [19, 20]. This method has been efficiently used for extracellular production of β-glucuronidase and amadoriase [21, 22]. This autolysis system has more advantages than most of the other methods, including its ease of operation and cost-effectiveness for extracellular protein production.

In this study, the secretion of LOX mediated by signal peptides, surfactants and the effects of bacteriophage lysis gene ΦX174-E on extracellular LOX production in *E. coli* were investigated. Our studies demonstrate a reference for engineering extracellular protein production.

## Results and discussion

### Secretory expression of LOX mediated by signal peptide in *E. coli*

**Fig. 1:**
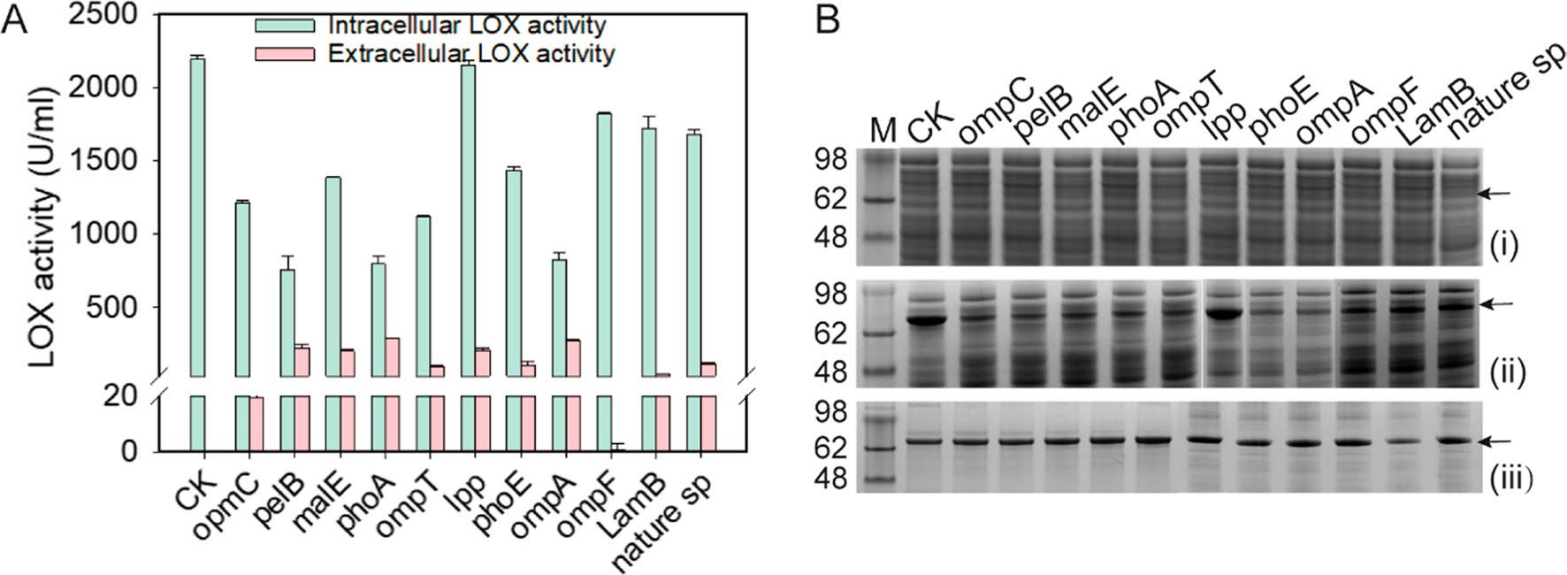
Effect of the signal peptide on the activity (**A**) and expression level (**B**) of lipoxygenase (LOX). SDS-PAGE analysis of fermentation broth supernatant (**i**), intracellular soluble proteins (**ii**), and intracellular insoluble proteins (**iii**)

The signal peptide is generally considered to be the first choice for recombinant protein secretion [9, 23]. First, we predicted the candidate signal peptides for LOX secretion using the SignalP 5.0 website (http://www.cbs.dtu.dk/services/SignalP/) (Table 1). All the selected signal peptides had obvious signal peptide cleavage peaks, indicating that they were potential signal peptides conducive to protein secretion. Then we constructed these recombinant expression plasmids carrying the corresponding signal peptides. After 36 h of fermentation, signal peptides were able to assist LOX secretion, including native signal peptide (from LOX of *Pseudomonas aeruginosa*, SP-LOX), SP-pelB, SP-MalE, SP-PhoA, SP-OmpA, and SP-Lpp, and the highest extracellular LOX activity was up to 288 U·mL^−1^ (Fig. 1A).

**Table 1:** Signal peptides used for LOX secretion

Signal peptide	Amino acid sequence
Nature signal	MKRRSVLLSGVALSGTALA
PelB (pectate lyase B) from Erwinia carotovora	MKYLLPTAAAGLLLLAAQPAMA
OmpA (outer-membrane protein A)	MKKTAIAIAVALAGFATVAQA
PhoA (alkaline phosphatase)	MKQSTIALALLPLLFTPVTKA
OmpF (outer-membrane protein F)	MMKRNILAVIVPALLVAGTANA
PhoE (outer-membrane pore protein E)	MKKSTLALVVMGIVASASVQA
MalE (maltose-binding protein)	MKIKTGARILALSALTTMMFSASALA
OmpC (outer-membrane protein C)	MKVKVLSLLVPALLVAGAANA
Lpp (murein lipoprotein)	MKATKLVLGAVILGSTLLAG
LamB (λ receptor protein)	MMITLRKLPLAVAVAAGVMSAQAMA
OmpT (protease VII)	MRAKLLGIVLTTPIAISSFA

Subsequently, the more intracellular LOX could be detected in physically broken cells and the highest was 2150.3 U·mL^−1^ (Fig. 1A). Compared with intracellular LOX activity, signal peptide-mediated extracellular production capacity was insufficient. Sodium dodecyl sulfate–polyacrylamide gel electrophoresis (SDS-PAGE) analysis also demonstrated that more LOX still accumulated in the cell and that it formed some inclusion bodies (Fig. 1B-iii). The LOX expression level mediated by the SP-Lpp signal peptide was similar to that of CK (without signal peptide), and other signal peptides reduced LOX expression levels. These results indicated that the secretory expression mediated by selected signal peptides could promote the extracellular production of LOX, but its secretion ability needed to be improved.

In gram-negative bacteria, Types I–VII secretion pathways enable the transfer of proteins across the cell envelope [24]. The type II secretion pathway is the most widely used in *E. coli* [14], and it consists of two processes: the (1) translocation of polypeptides containing a signal sequence in an unstructured state using the common Sec pathway into the periplasmic space; (2) processing into a mature protein with the assistance of molecular chaperones. The target protein subsequently enters the fermentation broth through active transport or non-specific leakage [14]. In this study, 11 different signal peptides involved in the type II pathway were tested for LOX secretion expression in *E. coli*. The protein localization analysis showed that more LOX accumulated in the periplasmic space than extracellular environment (Additional file 1: Fig. S1). However, from these results, it was established that some signal peptides significantly affected LOX expression levels (Fig. 1B-ii and B-iii). This may be because the signal peptides not only guided transmembrane transport but also affected protein translation [25]. For example, the interaction time with ectopic molecules can affect processing after protein synthesis, including folding, binding of chaperone molecules, and integration of transmembrane regions [26, 27]. Furthermore, the efficiency of protein secretion depends on the host strain, signal sequence, and target protein [13]. The reduced autolytic activity of the outer membrane, inefficient protein export system, and special characters of different secreted protein indicate that the signal peptides cannot always ensure the efficient secretion of recombinant proteins [9]. Currently, with the lack of general rules, it is still difficult to choose the appropriate signal peptide for target protein secretion [9].

### Effects of surfactants on the extracellular production of LOX

**Fig. 2:**
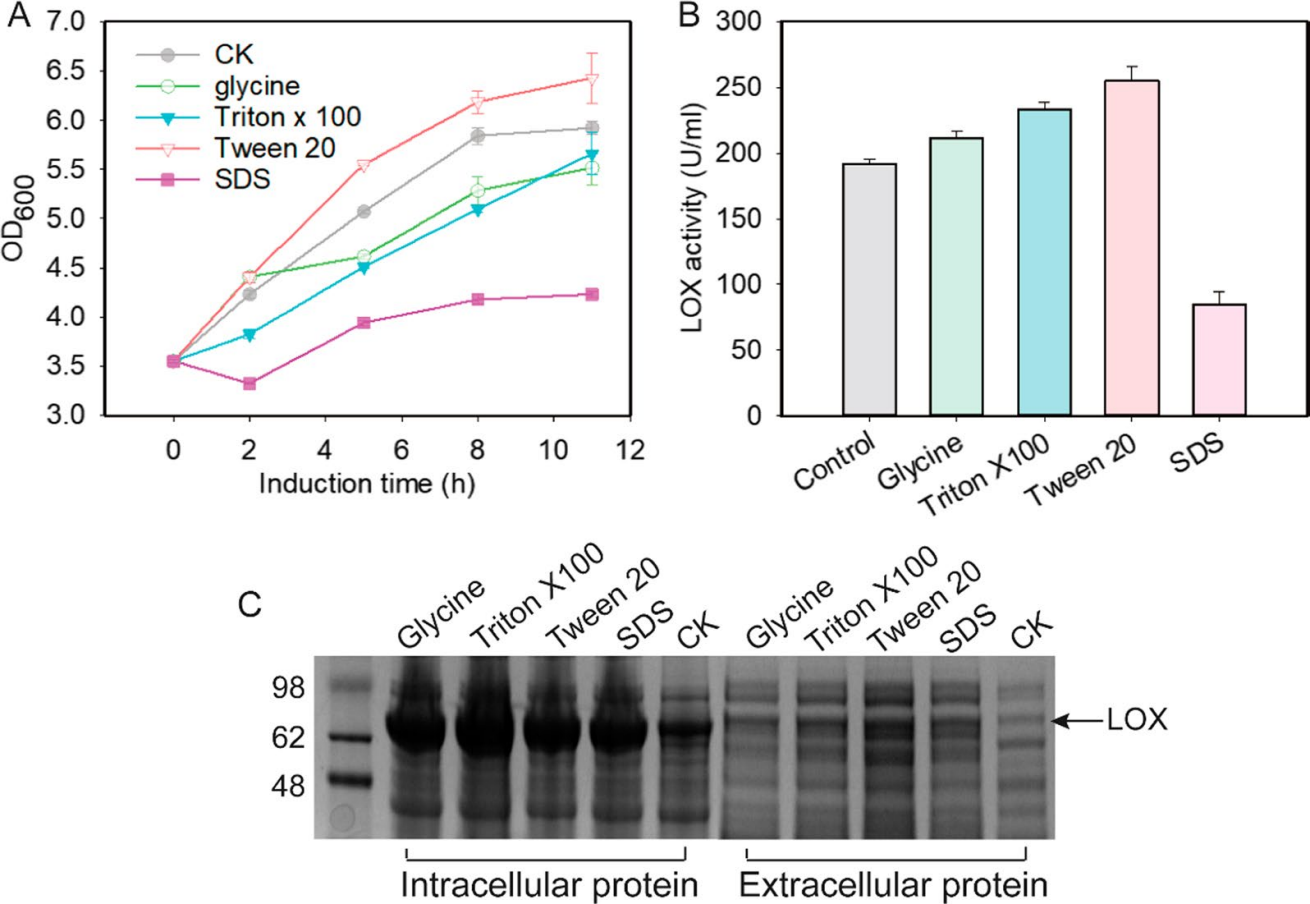
Effect of the surfactants and glycine on cell growth (**A**), extracellular activity (**B**), and LOX expression level (**C**). These substances contained Tween 20, TritonX-100, SDS, and glycine. OD_600_ represents cell density. Induction time was the time of lysis protein expression after adding arabinose

Generally, some substances affecting the channel proteins activity in the cell membrane, such as Tween 20, TritonX-100, SDS, and glycine, can improve the secretion efficiency and increase the permeability of the cell membrane by destroying the phospholipid bilayer to promote the extracellular production of recombinant proteins [28]. As the SP-Lpp signal peptide did not affect LOX expression levels, the SP-Lpp mediated LOX secretory expression was selected and optimization experiments using different surfactants or glycine were conducted. Thereafter, 0.5% (v/v) Tween-20, 0.5% (v/v) Triton X-100, 0.05% (w/v) SDS, and 0.5% (w/v) glycine were added into the culture medium and cell growth and LOX expression were analyzed. These results suggested that cell growth was only affected by the addition of SDS, and other surfactants and glycine slightly influenced cell growth (Fig. 2A). The LOX activity assay revealed that the addition of Tween20 effectively increased extracellular enzyme activity to 255.2 U·mL^−1^, which was 33% higher than that of the control group (Fig. 2B). Our results also demonstrated that the permeability of the cell membrane could be increased by the addition of surfactants, thereby contributing to the release of intracellular LOX into the fermentation broth. However, low extracellular LOX expression levels demonstrated that most of the LOX was still present in the cell even after the addition of surfactants (Fig. 2C) and therefore failed to meet the requirements for extracellular LOX production.

### Construction of an *E. coli* autolysis system and verification of extracellular production capacity

**Fig. 3:**
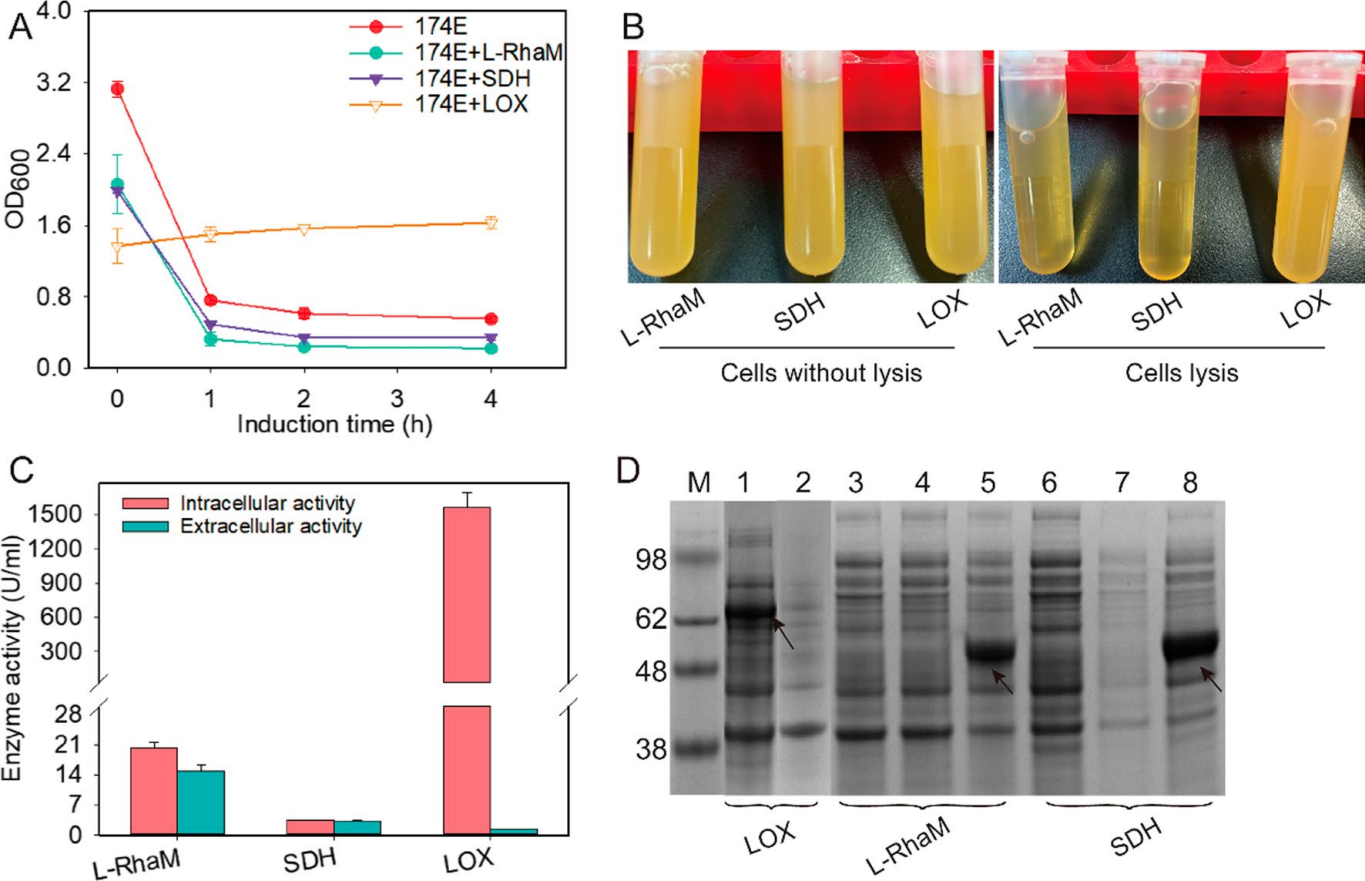
Different extracellular proteins produced by *E. coli* autolysis. **A** Time profile of cell growth after inducing cell lysis of autolytic *E. coli* that expressed different proteins. **B** Fermentation broth before and after cell lysis. **C** Extracellular enzyme activity after cell lysis. The red bar represents the intracellular enzyme activity after sonication. The green bar represents the extracellular enzyme activity by cell autolysis. **D** The expression level of extracellular protein after cell lysis. Lane 1 represents whole cells fraction; lanes 4 and 7 represent intracellular fraction; lanes 2, 5, and 8 represent fermentation broth supernatant; and lanes 3 and 6 represent cell debris after lysis. The arrow indicates the target protein

Due to the low efficiency of LOX secretion mediated by signal peptides, an autolysis system of *E. coli* was constructed for improved extracellular LOX production. Gene E from bacteriophage ΦX174, encoding a polypeptide with 91 amino acids, causes the lysis of *E. coli* following phage or heterologous expression [29]. Thus, here, the expression of ΦX174-E was regulated by the arabinose operon in *E. coli* BL21-174E to study extracellular production of the target protein. Then, the autolytic *E. coli* BL21 system was used for extracellular LOX production. In subsequent analyses, LOX without the signal peptide was selected because cytoplasmic proteins were more suitable for the autolysis system than the proteins secreted into the periplasmic space due to signal peptides. Our results showed that the cell density did not change when the lysis gene was induced for expression, which indicated that growth rate was consistent with the cell lysis rate (Fig. 3A). There was no visible change in transparency of fermentation broth after the induction of lysis protein (Fig. 3B). This may be because the function of lysis protein was inhibited by the potential interaction between LOX and cell membrane. Moreover, the difference in intracellular and extracellular enzyme activity was significant for the recombinant strain expressing LOX. In contrast to high intracellular LOX activity (1565 U·mL^−1^), minor extracellular LOX activity (1.5 U·mL^−1^) could be detected after the inducer was added (Fig. 3C).

To determine the function of the developed autolysis system, α-l-rhamnosidase (L-RhaM) from *Aspergillus nidulans* and l-sorbosone dehydrogenase (SDH) from *Gluconobacter oxydans* were selected for extracellular protein production under the same expression and cell autolysis condition. It is notable that the density of cells expressing L-RhaM and SDH decreased rapidly when arabinose was added to begin autolysis (Fig. 3A). The fermentation broth had a translucent and sticky appearance after lysis (Fig. 3B), which was caused by the release of cytosolic and nuclear molecules. The activity of L-RahM and SDH detected intracellularly before inducing autolysis was similar to that found extracellularly after autolysis induction (Fig. 3C), which suggested that target proteins were released into the medium as active forms. Protein expression levels were also analyzed and showed that L-RahM or SDH was not detected in soluble extract of the mechanically disrupted cells nor in the cell debris, and the fermentation supernatant showed clear target protein bands because autolysis released most of the cytoplasmic content into the culture medium (Fig. 3D). However, when comparing these results to those of LOX studies, it was necessary to optimize the lysis conditions for autolysis to promote the release of intracellular LOX.

### Effect of lysis conditions on the extracellular expression of LOX in autolytic *E. coli*

**Fig. 4:**
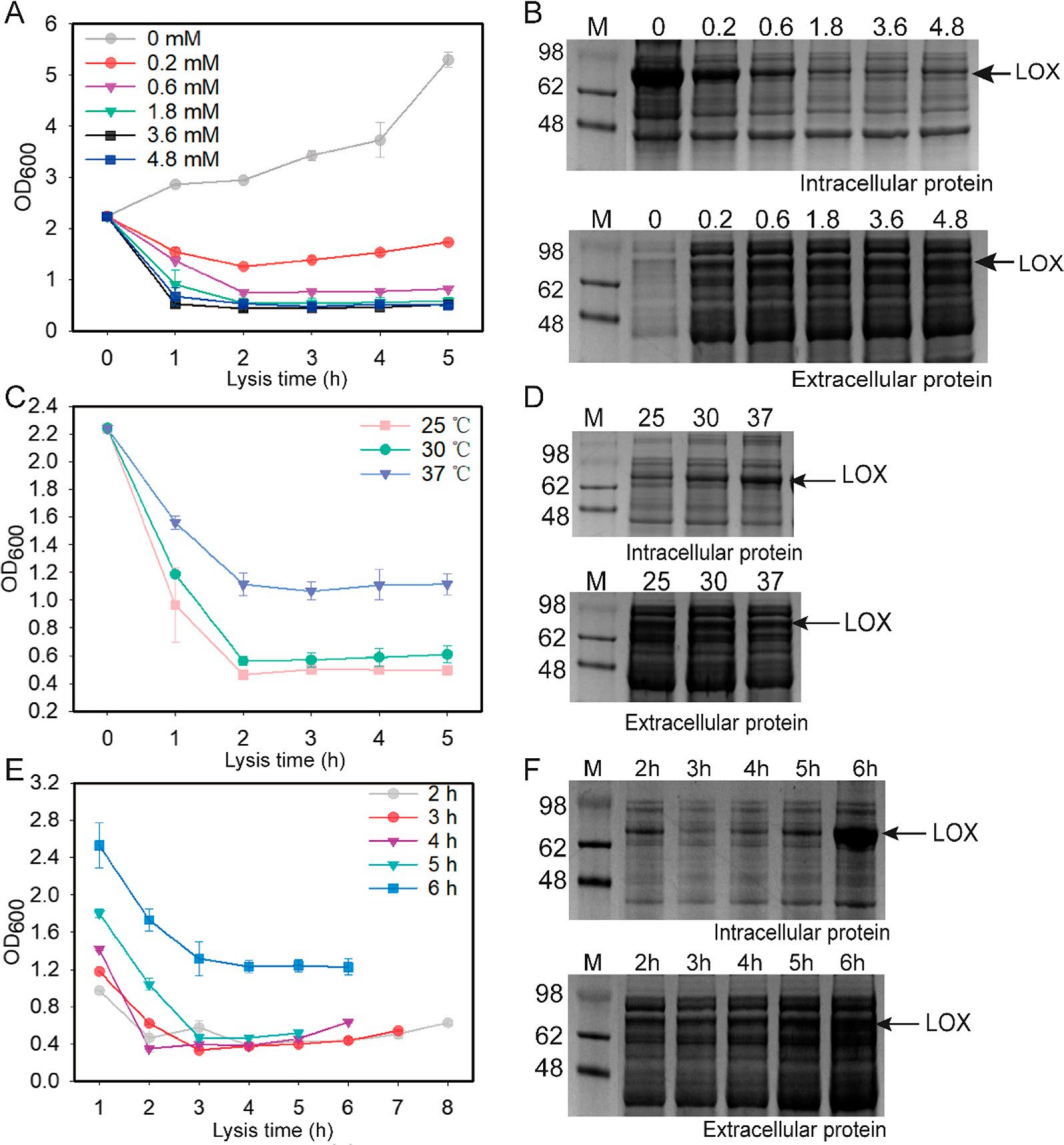
Effect of lysis conditions on the extracellular production of LOX in autolytic *E. coli*. Effect of inducer concentration (**A**), temperature (**C**), and induction time (**E**) on the growth of autolytic *E. coli*. Effect of inducer concentration (**B**), temperature (**D**), and induction time (**F**) on expression level of LOX in autolytic *E. coli*

To promote cell lysis and extracellular LOX production, the effects of arabinose concentration (0.2, 0.6, 1.8, 3.6, and 4.8 mM) on cell lysis were first investigated. The results showed that cell density rapidly decreased to below 0.5 in 2 h, when arabinose concentration was higher than 0.6 mM (Fig. 4A), the fermentation broth become clear and viscous. The protein expression levels of intracellular and extracellular LOX were further analyzed using SDS-PAGE and western blotting, and it was shown that LOX was effectively released when the arabinose concentration was higher than 0.6 mM (Fig. 4B and Additional file 1: Fig. S2). Therefore, the concentration of 0.6 mM arabinose was selected for cell lysis in the follow-up experiments.

Culture temperature also has a significant influence on protein expression and secretion. Therefore, here, the effects of induction temperature on cell lysis and extracellular LOX production were investigated. The lysis protein ΦX174-E was induced by 0.6 mM arabinose after LOX treatment for 6 h at 25 °C and then the autolysis strain was cultured at 25, 30, and 37 °C. These results demonstrated that the cell density at all temperatures significantly decreased after arabinose induction for 2 h. It is notable that the cell density at 37 °C was higher than at other temperatures, possibly because the lysis efficiency of ΦX174-E was insufficient to lyse all cells at higher temperatures (Fig. 4C). The protein expression levels were further analyzed using SDS-PAGE, which showed that the extracellular LOX expression level decreased with an increase in induction temperature. These results indicated that 25 °C was more conducive to active LOX expression and secretion (Fig. 4D).

To achieve a superior LOX yield, the time node between LOX expression and cell lysis was investigated. Recombinant *E. coli* expressing LOX was induced at 25 °C for 2–6 h and then 0.6 mM arabinose was added to induce expression of the lysis protein ΦX174-E. These results showed that cell density (OD_600_) could decrease to 0.4 approximately after the induction of ΦX174-E expression for 2 h (Fig. 4E). When cells were cultured for no more than 5 h, the OD_600_ decreased rapidly to approximately 0.5 after cell lysis was induced. However, after 6 h, the OD_600_ decreased slowly to approximately 1.3 following cell lysis. In addition, the analysis of extracellular/intracellular LOX activity showed that LOX was effectively released into the medium when the cells were cultured for less than 6 h, indicating that cell lysis might be affected by expression level of LOX or its potential interaction with components of cell membrane (Fig. 4F).

### Orthogonal optimization of lysis conditions and scale up in a 5-L fermenter

Currently, the orthogonal experimental method has been widely applied to optimize fermentation technical conditions in industrial fields [30–32]. Based on the results of our cell lysis experiments in autolytic *E. coli*, an orthogonal experiment of L^9^(4^3^) was designed for the following three influencing factors: the induction time for LOX, the concentration of the inducer (arabinose), and induction temperature for the lysis protein. However, the orthogonal design and analysis showed that these three factors had no significant effects on extracellular LOX production in autolytic *E. coli* (Table 2 and Additional file 1: Table S3). Considering the changes in cell density, the optimal conditions were A2B1C2, that is, an induction time and temperature of 5 h and 25 °C, respectively, and the concentration of arabinose was 0.6 mM.

**Table 2:** Orthogonal experiment design

Experiment number	LOX induction time (h) (A)	Induction temperature (°C) (B)	Arabinose concentration (g·L^−1^)(C)	Blank (D)	Cell density (OD_600_)
1	4	25	0.2	0	0.332 ± 0.05
2	4	30	0.6	0	0.214 ± 0.02
3	4	37	1.8	0	0.436 ± 0.04
4	5	25	0.6	0	0.302 ± 0.01
5	5	30	1.8	0	0.413 ± 0.06
6	5	37	0.2	0	1.134 ± 0.08
7	6	25	1.8	0	0.201 ± 0.03
8	6	30	0.2	0	0.869 ± 0.08
9	6	37	0.6	0	1.343 ± 0.09
K_m1_^a^	0.327	0.278	0.778		
K_m2_^a^	0.616	0.499	0.620		
K_m3_^a^	0.804	0.971	0.350		
S_m_^b^	0.477	0.693	0.428		

^a^K_mx_ is the average targeting value of each factor and can be expressed as K_mx_ = G_mx_/k_x_, where x (x = 1, 2, 3, 4, 5, 6) and m (m = A, B, C) are the level number and the factor, respectively, G_mx_ is the sum of the targeting indexes of all levels in each factor m, and k_x_ is the total level of the corresponding factor

^b^The range of factor S_m_ is calculated using S_m_ = max(K_mx_) − min(K_mx_)

**Fig. 5:**
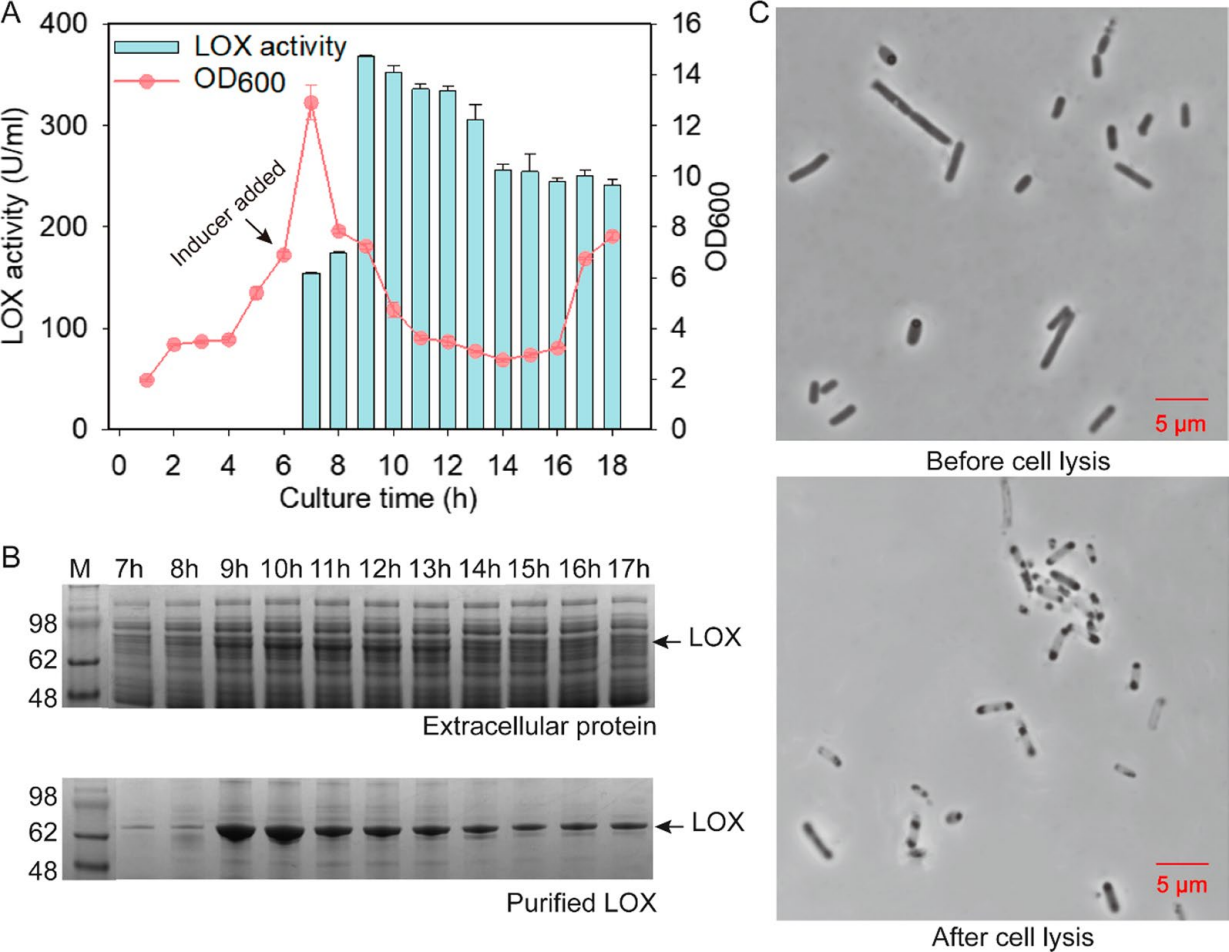
Characterization of cell growth, morphology, and extracellular LOX production in a 5-L fermenter. **A** Time profile of cell growth and LOX activity during the fermentation. **B** SDS-PAGE analysis of extracellular LOX in fermentation broth before and after purification. **C** Cell morphology of autolytic *E. coli* before and after cell lysis

To test the autolysis system, we conducted a scaled-up culture of the extracellular production of LOX in a 5-L fermenter. First, the induction time culture for expression (3, 4, 5, 6, and 7 h) was optimized. We found that the expression levels of LOX increased with the culture time prolonged, while the cells could not lysis when the culture time was more than 7 h. Thus, the selected optimal induced time was 6–7 h in the fermenter (Additional file 1: Figs. S3A and S3B). Subsequently, the cell density of autolyzed *E. coli* was 6.9 approximately 6 h after inducing LOX expression in the self-induction medium, and 0.6 mM arabinose was added to induce lysis. Cell growth and LOX expression levels were evaluated throughout the fermentation process. Arabinose was added to induce cell lysis after culturing for 6 h, and cell density decreased rapidly after 1 h of cell lysis and achieved the lowest levels (OD_600_ = 2.7) at 8 h after cell lysis. Moreover, LOX activity elevated with the decline in cell density, and the highest activity reached 368 ± 1.4 U·mL^−1^ after 9 h of culture (Fig. 5A). Furthermore, the fermentation broth had a viscous appearance after lysis. The analysis of LOX expression levels at different lysis time showed that the extracellular LOX protein bands appeared after 7 h of culture. To avoid interference from the cell lysates in the fermentation broth, LOX was further purified and analyzed through the same volume of fermentation broth at different fermentation stages, and the extracellular LOX expression levels reached maximum levels after 9–10 h of culture (Fig. 5B). Morphological changes in cell lysis processes were observed using a microscope, and we found that cells that had been lysed were rod-shaped and empty (Fig. 5C). We also observed protein aggregation at the edges of the empty cells, which might be caused by LOX inclusion bodies in cells. The above results indicated that the autolysis *of E. coli* could effectively promote the extracellular production of LOX.

In this study, we obtained extracellular LOX production by inducing the expression of the ΦX174-E lysis gene. An arabinose promoter was used to express the heterologous lysis protein to achieve extracellular LOX production. Cells could not be lysed after LOX overexpression more than 6 h. This may be because ΦX174-E protein-mediated cell lysis is affected by several factors, such as lipid composition, cytoplasmic membrane, and the activity of the autolysis system [29, 33]. For example, LOX can interact with phospholipids in the cell membrane, which changes the outer and inner membrane protein patterns and limits the degradation of peptidoglycan [34, 35]. In addition, the lysis of bacteria caused by ΦX174-E perforin depends on the growth state of bacteria, culture medium, pH value, Mg^2+^ concentration, and the fluidity of the membrane [36]. Therefore, the control of the cell growth state and culture conditions before lysis are critical for cell lysis and the efficient production of extracellular LOX.

In our previous studies, inclusion bodies of LOX formed at different induction temperatures (Additional file 1: Fig. S5). In this study, the content of inclusion bodies decreased after cell lysis, indicating that the timely release of the target protein might relieve translation stress and promote soluble protein expression. Although the changes in enzymatic activity did not precisely correspond to the amount of the target protein in the crude enzyme (Fig. 5A and B), it was found that enzyme activity and protein concentration were roughly identical in purified LOX (Fig. 5B and Additional file 1: Fig. S4). Therefore, it was speculated that potential LOX inhibitors might interfere with the determination of LOX activity in the crude enzyme.

## Conclusion

In this study, multiple strategies including signal peptides, surfactants, and cell autolysis system based on bacteriophage lysis gene ΦX174-E were developed and characterized for extracellular LOX production. By comparison, the highest yield of extracellular LOX in *E. coli* was up to 368 ± 1.4 U·mL^−1^ under the optimized autolysis and fermentation conditions. Furthermore, the underlying relationships between LOX overexpression and cell autolysis still need further analysis, which would be helpful to improve extracellular LOX production.

## Materials and methods

### Strains, plasmids, and culture conditions

The strains, plasmids, and primers used in this study are listed in Additional file 1: Tables S1 and S2. *Escherichia coli* JM109, used as the cloning host for plasmid construction, was grown in Luria–Bertani (LB) broth (5 g·L^−1^ yeast extract, 10 g·L^−1^ tryptone, and 10 g·L^−1^ NaCl), with 1.5% Bacto agar. It was used as the expression strain in the self-induction medium (12 g·L^−1^ tryptone, 24 g·L^−1^ yeast extract, 6 g·L^−1^ glycerol, 2.31 g·L^−1^ KH_2_PO_4_, 16.84 g·L^−1^ K_2_HPO_4_, 0.5 g·L^−1^ glucose, and 1.5 g·L^−1^ lactose), modified from Terrific Broth medium (12 g·L^−1^ tryptone, 24 g·L^−1^ yeast extract, 5 g·L^−1^ glycerol, 2.31 g·L^−1^ KH_2_PO_4_, and 16.84 g·L^−1^ K_2_HPO_4_), for fermentation. Ampicillin and streptomycin were added to the medium with a final concentration of 100 μg·mL^−1^ to maintain the plasmid.

### Plasmid construction

pET22b (+)/LOX had been previously constructed in the laboratory to express LOX in *E. coli* BL21 (DE3) [8]. The *lox* gene was from *Pseudomonas aeruginosa.* Different signal peptide sequences were searched for in scientific reports. Based on the prediction of the SignalP 5.0, the potential signal peptide sequence was cloned into the N-terminal of the LOX sequence of the plasmid pET22b (+)/LOX. Then, pET22b (+)/sps-LOX, containing different signal peptide sequences, was obtained through colony PCR and sequencing.

The bacteriophage lysis gene (ΦX174-E) fragment, synthesized at Sangon Biotech (Shanghai, China), was inserted between the restriction sites NcoI and HindIII of the pBAD plasmid, and the plasmid pBAD-E was obtained after sequencing. Using pCDF Duet-1 as the plasmid template, the pCDF Duet-1 vector fragment was amplified using primers pCDFE-F and pCDFE-R. The expression box fragment containing the arabinose operon araBAD promoter and bacteriophage lysis gene ΦX174-E were amplified using primers pBAD-F and pBAD-R. After one-step of cloning, colony PCR, and sequencing, the pCDF/araBAD-E plasmid that was strictly regulated by arabinose was successfully obtained.

### Expression of LOX, L-RhaM and SDH in flasks

To express LOX, an overnight culture of recombinant *E. coli* LOX-E strains in LB medium was sub-cultured in a self-induction medium at 37 °C and 220 rpm. The fermentation temperature was decreased to 25 °C when the cell density reached 0.6, and cells were cultured at 220 rpm for the expression of recombinant LOX. After 1–6 h of induction, arabinose was added to induce ΦX174-E expression. The method for estimating the number of cells and cell density is the use of optical density (OD) measurements at a wavelength of 600 nm (OD_600_). The expression and induction conditions of L-RhaM and SDH were the same as that of LOX.

### Production of LOX in a 5-L fermenter

The recombinant *E. coli* strains were used to scale up fermentation in a 5-L fermenter (Eppendorf NBS, USA) with 2.5 L self-induction medium, 100 µg·mL^−1^ of ampicillin, and streptomycin. A 10% (v/v); seed culture in LB medium was inoculated into the self-induction medium for cultivation. The stirring speed of fermentation was 400 rpm, and the ventilation volume was 2.5 vvm. After incubation at 37 °C for 2 h, the fermentation temperature was reduced to 25 °C. When strain density was cultured to an optical density at 600 nm (OD_600_) of 7, ΦX174-E expression was induced with 0.6 mM arabinose at 25 °C.

### Periplasmic space protein extraction

The LOX protein in periplasmic space was extracted using EDTA-sucrose gradient centrifugation. The specific process was as follows: the cells were collected using centrifugation at 6000×*g* at 4 °C for 10 min at the end of fermentation and then washed twice using ultrapure water. Subsequently, the cells were resuspended in the same volume of EDTA-sucrose solution (1 mM EDTA, pH 8.0; 25% sucrose, w/v). The supernatants were collected by centrifugation at 10,000×*g* for 30 min after immersion for 3 h in an ice bath, and this was taken as the periplasmic space protein.

### SDS-PAGE and protein concentration assay

Aliquots of the *E. coli* fermentation supernatant and whole cells (resuspended fraction) were mixed with SDS loading buffer (NuPAGE1 LDS Sample Buffer 4×, Fisher Scientific) at a ratio of 3:1, and SDS-PAGE was performed as previously described [37]. Using bovine serum albumin as a standard, the protein concentration was determined using the Bradford assay method [38].

### Western blot

To demonstrate the LOX band in fermentation broth after cell lysis, a western blot analysis was performed using the wet blot procedure [18]. After electrophoresis, the samples were transferred to a polyvinylidene fluoride (PVDF, 0.22 μm) membrane. Membranes were blocked overnight at 4 °C in blocking buffer containing 5% (w/v) nonfat milk powder and 0.1% (v/v) Tween20 in TBST buffer (20 mM Tris, pH 7.4, 150 mM NaCl). Then, membranes and primary antibody (mouse monoclonal anti-histidine tag [anti-His6, Clone AD1.1.10, Bio-Rad antibodies]) diluted in 2 mL of 5% nonfat milk powder/TBST were incubated at 4 °C for 6–8 h. It was then washed thrice for 5 min in ~ 20 mL TBST at 25 °C in a small box on a shaker. Subsequently, membranes and secondary antibody (goat anti-mouse IgG1-horseradish peroxidase conjugate [Bio-Rad antibodies]) at a 1:7000 dilution in blocking buffer were incubated at 25 °C for 1–2 h. Membranes were then washed thrice for 5 min in ~ 20 mL TBST. Protein was detected using chemiluminescence with the signal enhanced HRP-DAB substrate chromogenic Kit (TianGen, Beijing, China) according to the manufacturer’s protocols to detect bands.

### Purification of LOX from the recombinant *E. coli*

The LOX protein was purified using nickel column affinity chromatography. The specific process was as follows: the fermentation supernatant was collected by centrifugation at 12,000×*g* at 4 °C for 30 min after cell lysis, and then filtered using a 0.22-μm mixed cellulose membranes. Nickel affinity chromatography (using a His Trap TM FF crude column, GE Healthcare) was performed by washing the beads with 5–10 column volumes of distilled water to eliminate the preservatives and equilibrating the column with 5–10 column volumes of binding buffer (20 mM phosphate buffer, 20 mM K_2_HPO_4_ and KH_2_PO_4_, pH 7.4). The sample was loaded into a nickel affinity chromatography column pre-equilibrated with buffer A (20 mM phosphate buffer, 20 mM K_2_HPO_4_, and KH_2_PO_4_, pH 7.4). After re-equilibration, the hetero protein was eluted with 15% buffer B (20 mM phosphate buffer, 20 mM K_2_HPO_4_ and KH_2_PO_4_, 500 mM imidazole, pH 7.4). The target LOX was eluted with 25% buffer B and then dialyzed overnight at 4 °C in pre-cooled buffer A to obtain pure protein. It was then necessary to concentrate the diluted desalinated LOX sample with a concentration tube for subsequent analyses.

### Assays for LOX activity

The catalytic activity of LOX was determined according to the methods described by Pang [8]. One unit of LOX activity is defined as a 0.001 increase in absorbance value in 1 min at 470 nm. The specific operation steps were as follows: Linoleic acid (LA) as the substrate was diluted in phosphate buffer (20 mM K_2_HPO_4_ and KH_2_PO_4_, pH 7.4) to a final concentration of 0.32 mM. Unless otherwise stated, 20 µL of diluted LOX solution was incubated with the LA stock solution for 4 min, in the presence of 20 mM phosphate buffer (pH 7.4) to a volume of 200 µL. The reactions were allowed to continue by the addition of 10 µL of 1% soluble starch and 40 µL of saturated potassium iodide solution to the mixed solution of enzyme and substrate every 20 s. The assay was completed by the addition of 500 µL of 15% acetic acid solution [39, 40]. In all experiments, we added inactive LOX to the samples as a blank to check for interference of other substances in the reaction system during the incubation period. Activities were measured in triplicate; error bars indicate the standard deviation from the mean.

## Supplementary Information

**Additional file 1. Fig. S1**: Secretory expression of LOX mediated by signal peptide in E. coli;** Fig. S2**: Effect of lysis conditions on the extracellular expression of LOX in autolytic E. coli;** Fig. S3**:Orthogonal optimization of lysis conditions and scale up in a 5-L fermenter;** Fig. S4**:Orthogonal optimization of lysis conditions and scale up in a 5-L fermenter;** Table S1**:Strains, plasmids, and culture conditions;** Table S2**: Strains, plasmids, and culture conditions;** Table S3**:Orthogonal optimization of lysis conditions and scale up in a 5-L fermenter.

## Data Availability

All data generated or analyzed during this study are included in this published article and its supplementary information files.
