# Supplementary material for: Enhancing extracellular production of lipoxygenase in Escherichia coli by signal peptides and autolysis system
Source: Microb Cell Fact. 2022 Mar 19;21:42. doi: 10.1186/s12934-022-01772-x (PMC8933919; doi:10.1186/s12934-022-01772-x)
Supplement: Supplementary file 1 — Additional file 1. Fig. S1: Secretory expression of LOX mediated by signal peptide in E. coli; Fig. S2: Effect of lysis conditions on the extracellular expression of LOX in autolytic E. coli; Fig. S3:Orthogonal optimization of lysis conditions and scale up in a 5-L fermenter; Fig. S4:Orthogonal optimization of lysis conditions and scale up in a 5-L fermenter; Table S1:Strains, plasmids, and culture conditions; Table S2: Strains, plasmids, and culture conditions; Table S3:Orthogonal optimization of lysis conditions and scale up in a 5-L fermenter. [file 12934_2022_1772_MOESM1_ESM.docx]

**Microbial Cell Factories**

**Development of signal peptides and autolysis system for extracellular preparation of lipoxygenase in *Escherichia coli***

Cuiping Pang^1,2^, Song Liu^1,2^, Guoqiang Zhang^1,2,4^*, Jingwen Zhou^1,2,4^, Guocheng Du^2,3^, Jianghua Li^2^

^1^National Engineering Laboratory for Cereal Fermentation Technology, Jiangnan University, 1800 Lihu Road, Wuxi, Jiangsu 214122, China

^2^School of Biotechnology and Key Laboratory of Industrial Biotechnology, Ministry of Education, Jiangnan University, 1800 Lihu Road, Wuxi, Jiangsu 214122, China

^3^The Key Laboratory of Carbohydrate Chemistry and Biotechnology, Ministry of Education, Jiangnan University, 1800 Lihu Road, Wuxi, Jiangsu 214122, China

^4^Jiangsu Provisional Research Center for Bioactive Product Processing Technology, Jiangnan University, 1800 Lihu Road, Wuxi, Jiangsu 214122, China

*Corresponding author: Guoqiang Zhang

Mailing address: School of Biotechnology, Jiangnan University, 1800 Lihu Road, Wuxi, Jiangsu 214122, China

Phone: +86-510-85914371

Fax: +86-510-85914371

E-mail: gqzhang@jiangnan.edu.cn

**Supporting Information**

**
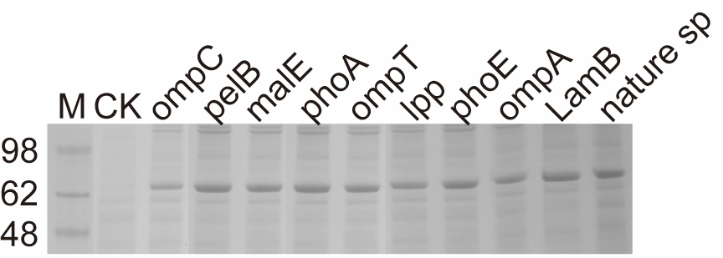
**

**Fig. S1 Expression level of target protein LOX in periplasmic space**

**
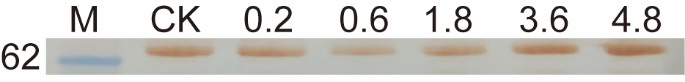
**

**Fig. S2 LOX in fermentation broth detected by western blot**


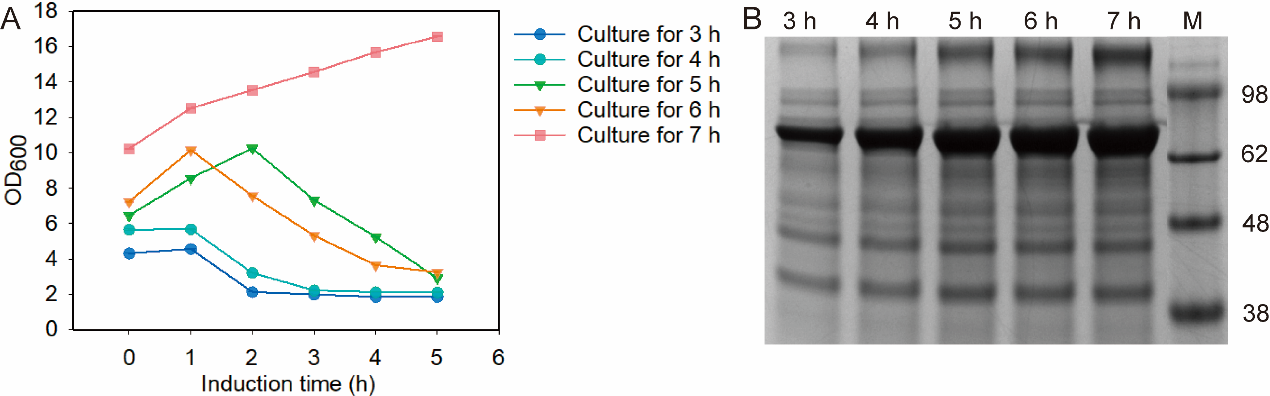


**Fig. S3 Optimization of induction time in 5-L fermentor.** A. Changes of OD_600_ after adding inducer at different culture times. B. LOX expression level at different culture time.


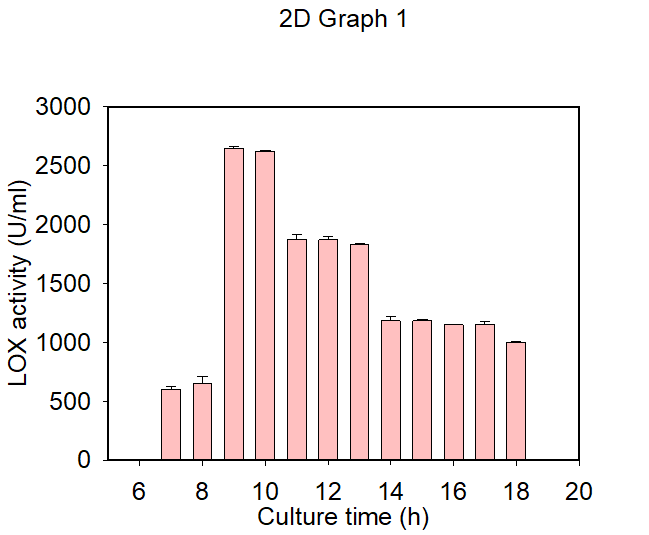


**Fig. S4 Purified LOX activity at different culture time**


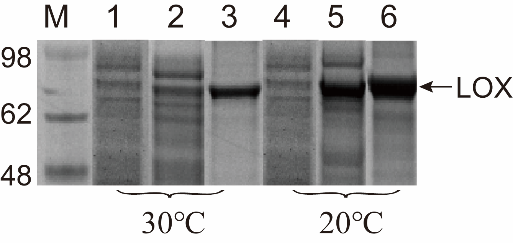


**Fig. S5 Effect of induction temperature on LOX expression level**

M: Marker; 1, 4: Extracellular protein expression level at different temperature; 2, 5: Expression level of intracellular soluble protein at different temperature; 3, 6: Expression level of intracellular insoluble protein at different temperature.

**Table S1 Plasmids and strains used in this work**

| Plasmid/strain | Relevant characteristics | Sources or references |
| --- | --- | --- |
| Plasmid |  |  |
| pCDF Duet-1 | Expression vector, Streptomycin resistance | Lab stock |
| pBAD | Expression vector carrying ParaBAD promoter and arabinose operon | Lab stock |
| pCDF/araBAD-E | pCDF carring ParaBAD promoter, arabinose operon and phage ΦX174-E lysis protein E | This work |
| pET22b(+)/sp-Pa*lox* | pET22b (+)/palox carring LOX natural signal peptide and pelB, phoe, Mal, OmpA, OmpC, OmpT, OmpF, PhoA, Lame, Lpp signal peptide, respectively. | This work |
| Strain |  |  |
| *E. coli* JM109 | The cloning host for plasmid construction | This work |
| *E. coli* BL21(DE3) | The expression host for protein expression | This work |
| pET22b(+)-parABS/LOX | *E．coli* BL21(DE3) carring plasmid *E. coli* BL21(DE3) carring pET22b(+)-parABS/LOX plasmid | Pang et al. (2020) |
| LOX-E | *E．coli* BL21(DE3) carring pET22b(+)-parABS/LOX and pCDF/araBAD-E plasmid | This work |

**Table S2 Primers used in this study**

| Primer | Sequence |
| --- | --- |
| pelB-F | gcagcagcggtcggcagcaggtatttcatCATATGtatatctccttcttaaagttaaacaaaattatttctagag |
| pelB-R | gctgccgaccgctgctgctggtctgctgctcctcgctgcccagccggcgatggccCATCATCATCATCATCATGCAGAAGCAG |
| phoA-F | gagtgccagtgcaatagtgctttgtttcacCATATGtatatctccttcttaaagttaaacaaaattatttctagag |
| phoA-R | cactattgcactggcactcttaccgttactgtttacccctgtgacaaaagccCATCATCATCATCATCATGCAGAAGCAG |
| ompA-F | gccactgcaatcgcgatagctgtctttttcatCATATGtatatctccttcttaaagttaaacaaaattatttctagag |
| ompA-R | cgcgattgcagtggcactggctggtttcgctaccgtagcgcaggccCATCATCATCATCATCATGCAGAAGCAG |
| ompF-F | gacgatcactgccagaatattgcgcttcatcatCATATGtatatctccttcttaaagttaaacaaaattatttctagag |
| ompF-R | tattctggcagtgatcgtccctgctctgttagtagcaggtactgcaaacgctCATCATCATCATCATCATGCAGAAGCAG |
| phoE-F | caatgcccatcaccactaatgccagagtgctctttttcatCATATGtatatctccttcttaaagttaaacaaaattatttctagag |
| phoE-R | cattagtggtgatgggcattgtggcatctgcatctgtacaggctCATCATCATCATCATCATGCAGAAGCAG |
| ompT-F | gttgtcaggactattcccagaagtttcgcccgcatCATATGtatatctccttcttaaagttaaacaaaattatttctagag |
| ompT-R | gaatagtcctgacaacccctattgcgatcagctcttttgctCATCATCATCATCATCATGCAGAAGCAG |
| malE-F | cggataatgcgaggatgcgtgcacctgtttttattttcatCATATGtatatctccttcttaaagttaaacaaaattatttctagag |
| malE-R | catcctcgcattatccgcattaacgacgatgatgttttccgcctcggctctcgccCATCATCATCATCATCATGCAGAAGCAG |
| ompC-F | gcagagctgggaccaggagggacagtactttaactttcatCATATGtatatctccttcttaaagttaaacaaaattatttctagag |
| ompC-R | ctggtcccagctctgctggtagcaggcgcagcaaacgctCATCATCATCATCATCATGCAGAAGCAG |
| lpp-F | actgggcgcggtaatcctgggttctactctgctggcaggtCATATGtatatctccttcttaaagttaaacaaaattatttctagag |
| lpp-R | caggattaccgcgcccagtaccagtttagtagctttcatCATCATCATCATCATCATGCAGAAGCAG |
| lamB-F | gttgccgtcgcagcgggcgtaatgtctgctcaggcaatggctCATATGtatatctccttcttaaagttaaacaaaattatttctagag |
| lamB-R | cgcccgctgcgacggcaaccgccagaggaagtttgcgcagagtaatcatcatCATCATCATCATCATCATGCAGAAGCAG |
| E-F | CAGGAGGAATTAACCATGGatggtacgctggactttgtgggatac |
| E-R | CGCCAAAACAGCCAAGCTTtcactccttccgcacgtaatttttgac |
| pBAD-F | cttacattaattgcgttgcgcGTTCCGCGCACATTTCCCCGAAAAG |
| pBAD-R | cacaaagtccagcgtaccatTTTTTATAACCTCCTTAGAGCTCGAATTC |
| pCDFE-F | atggtacgctggactttgtgggataccctc |
| pCDFE-R | gcgcaacgcaattaatgtaagttagctc |

**Table S3 Analysis of variance**

| parameter | Sources of variance | S^a^ | f^b^ | F^c^ | Fa^d^ |
| --- | --- | --- | --- | --- | --- |
| OD_600_ | LOX induction time | 0.346 | 2 | 0.753 | F 0.01(2,4)=10.9 |
|  | Induction temperature | 0.751 | 2 | 1.635 | F 0.05(2,4)=5.14 |
|  | Arabinose concentration | 0.281 | 2 | 0.612 |  |
|  | T^e^ | 1.38 | 6 |  |  |

S*^a^* is sum of mean deviation squares. f*^b^* is free degree. F*^c^*: The ratio of the sum of the squares of the average deviation caused by the change of the factor level to the sum of the squares of the average deviation of the error. Fa*^d^*: Critical value of F-test, for example, in F_0.01(2,4)，_ 0.01, 2 and 4 is reliability, free degree and free degree of error, respectively. T*^e^* is sum of mean square deviation of error.

**Reference**

Pang, C., Liu, S., Zhang, G., Zhou, J., Du, G., Li, J. 2020. Combinatorial strategy towards the efficient expression of lipoxygenase in Escherichia coli at elevated temperatures. *Appl Microbiol Biotechnol*, **104**(23), 10047-10057.
